# Increased arterial stiffness in healthy subjects with high-normal glucose levels and in subjects with pre-diabetes

**DOI:** 10.1186/1475-2840-10-30

**Published:** 2011-04-15

**Authors:** Jin Young Shin, Hye Ree Lee, Duk Chul Lee

**Affiliations:** 1 Department of Family Medicine, Gangnam Severance Hospital, Yonsei University College of Medicine, Seoul, Korea; 2 Department of Family Medicine, Severance Hospital, Yonsei University College of Medicine, Seoul, Korea

## Abstract

**Background:**

Increased fasting plasma glucose (FPG), which includes impaired fasting glucose (IFG), impaired glucose tolerance (IGT), and diabetes, is a risk factor for arterial stiffness. While IFG is widely accepted as a cardiovascular risk factor, recent studies have argued that subjects with high-normal glucose level were characterized by a high incidence of cardiovascular disease. The purpose of this study is to investigate the relationship between FPG and arterial stiffness in non-diabetic healthy subjects.

**Methods:**

We recruited 697 subjects who visited the health promotion center of a university hospital from May 2007 to August 2008. Age, sex, body mass index (BMI), resting heart rate, smoking habits, alcohol intake, exercise, blood pressure, medical history, FPG, lipid profile, high sensitivity C-reactive protein (hs-CRP), and Brachial-ankle pulse wave velocity (ba-PWV) were measured. We performed correlation and multiple linear regression analyses to divide the research subjects into quartiles: Q1(n = 172), 65 mg/dL ≤FPG < 84 mg/dL; Q2(n = 188), 84 mg/dL ≤FPG < 91 mg/dl; Q3(n = 199), 91 mg/dL ≤FPG < 100 mg/dL; Q4(n = 138), 100 mg/dL ≤FPG < 126 mg/dL.

**Results:**

FPG has an independent, positive association with ba-PWV in non-diabetic subjects after correcting for confounding variables, including age, sex, BMI, blood pressure, resting heart rate, hs-CRP, lipid profile, and behavioral habits. The mean ba-PWV of the high-normal glucose group (Q3, 1384 cm/s) was higher than that of the low-normal glucose group (1303 ± 196 cm/s vs.1328 ± 167 cm/s, P < 0.05). The mean ba-PWV value in the IFG group (1469 ± 220 cm/s) was higher than that in the normoglycemic group (P < 0.05, respectively).

**Conclusions:**

An increase in FPG, even within the normal range, was associated with aggravated arterial stiffness. Further research is needed to determine the glycemic target value for the prevention of arterial stiffness in clinical and public health settings.

## Background

Increased fasting plasma glucose (FPG), which includes impaired fasting glucose (IFG), impaired glucose tolerance (IGT), and diabetes, is a risk factor for arterial stiffness and cardiovascular disease[1-4]. Because there is no threshold glucose level to indicate the development of vascular disease, determining the appropriate target FPG level for the prevention of cardiovascular risk is a topic of active research[5-7]. While IFG is widely accepted as a cardiovascular risk factor, recent studies have demonstrated that subjects with high-normal glucose level are characterized by a high incidence of cardiovascular disease[8-10]. This finding indicates that, even in the normal range, increased FPG can be associated with aggravation of arterial stiffness, which is considered an early marker of atherosclerosis.

Arterial stiffness can be easily and noninvasively assessed by measuring the pulse wave velocity (PWV)[11,12]. Brachial-ankle PWV (ba-PWV) is a suitable measure for screening for vascular dysfunction and the development of atherosclerosis in a preventive setting [13,14]. We assessed whether the FPG level is associated with arterial stiffness by measuring ba-PWV in non-diabetic healthy subjects with no history of cardiovascular disease, hypertension, or dyslipidemia.

## Methods

### Study Population

We recruited 856 non-diabetic subjects who visited the health promotion center of a university hospital from May 2007 to August 2008. We excluded 152 subjects with history of anti-diabetes medication; anti-hypertensive medication; lipid-lowering medication; stroke; or cardiovascular disease, such as coronary heart disease, peripheral arterial disease, arrhythmia, congestive heart failure, or valvular heart disease. We also excluded six subjects who had extremely low FPG levels (50-65 mg/dL) and one subject who had a low ankle-brachial index (ABI) (<0.9), which suggested the subject might have had peripheral arterial occlusive disease. After the exclusions, 697 subjects were included in this study. The institutional review board of Gangnam Severance Hospital, Yonsei University College of Medicine approved this study, and informed consent was obtained from each participant.

The study sample was divided into four groups according to FPG level. Using the diagnostic criteria of the American Diabetes Association [15], after grouping the IFG subjects (Q4, 100 mg/dL ≤FPG<126 mg/dL), FPG levels were categorized into quartiles: Q1(n = 172), 65 mg/dL ≤FPG<84 mg/dL; Q2(n = 188), 84 mg/dL ≤FPG<91 mg/dl; and Q3(n = 199), 91 mg/dL ≤FPG<100 mg/dL, Q4(n = 138):100 mg/dL ≤FPG<126 mg/dL. Blood pressure and resting heart rate were measured after resting for more than five minutes. Anthropometric measurements were used to calculate the body mass index (BMI). To reduce inter-observer variation in measurements, one researcher measured all anthropometric parameters throughout the study. A questionnaire was used to obtain information about participant medical history and lifestyle, such as exercise, smoking habits, and alcohol ingestion. Subjects were instructed to refrain from alcohol on the day before testing and from smoking, coffee, tea, and pain medication on the day of measurement.

After an overnight fast, serum glucose, total cholesterol and high density lipoprotein (HDL)-cholesterol levels were measured via enzymatic procedures using an autoanalyser (Bayer, Terrytown, NY, USA). Non-high density lipoprotein (HDL) cholesterol was calculated by subtracting HDL-cholesterol from total cholesterol. Non-HDL cholesterol includes all known potential atherogenic lipid particles. All variables were adjusted according to non-HDL cholesterol because it is a useful predictor of risk for cardiovascular disease[16,17]. High-sensitivity C-reactive protein (hs-CRP) was measured using a latex-enhanced immunoturbidimetric assay in an ADVIA 1650 Chemistry System (Bayer).

### PWV Measurement

The ba-PWV was measured using a volume plethysmographic instrument (PWV/ABI, Colin Co, Komaki, Japan). This device records a phonocardiogram, electrocardiogram, volume pulse form, and arterial blood pressure at the left and the right brachial arteries and ankles. The ba-PWV was calculated using time-phase analysis between the right brachial artery pressure and the volume waveforms at both ankles. The distance between the right brachium and the ankle was estimated based on the subject's height. We used the mean ba-PWV in analyses because the right and the left ba-PWV are significantly correlated[18]. Both ba-PWV values were measured after allowing the patient to rest in the supine position for at least five minutes in an air-conditioned room (24 to 26°C). The validity, reliability, and reproducibility of this instrument were assessed in a previous study [19].

### Statistical Analysis

SAS 9·1 was used for statistical analyses (SAS Institute, Cary, NC, USA). Mean values of the clinical characteristics were compared among the four subject groups using one-way analysis for continuous variables and the Chi-squared test for categorical variables. Because of the skewed distribution of hs-CRP, this parameter was logarithmically transform; however, untransformed raw data are presented for the mean ± standard deviation (SD) in tables. Pearson's correlation coefficients were calculated to evaluate the relationships between ba-PWV and clinical variables. After adjusting for age, sex, blood pressure (systolic/diastolic), body mass index, resting heart rate, hs-CRP, HDL-cholesterol, non-HDL cholesterol, exercise, smoking, and alcohol ingestion, a multiple linear regression analysis was performed to identify any independent associations between ba-PWV and FPG level. The mean ba-PWV of the quartile was analyzed using an ANCOVA test considering confounding factors. *P *values less than 0.05 were considered statistically significant.

## Results

The characteristics of the 697 subjects enrolled in this study are shown in **Table **1. The subjects were adults between 20 and 79 years of age, with a mean age of 52.3 ± 9.1 years. The mean glucose levels were as follows: Q1 (77.8 ± 4.2 mg/dl), Q2 (87.2 ± 2.0 mg/dl), Q3 (94.7 ± 2.5 mg/dl), and Q4 (107.0 ± 6.2 mg/dl). In addition, BMI, systolic blood pressure, diastolic blood pressure, hs-CRP, resting heart rate, non-HDL cholesterol, male: female ratio, and alcohol ingestion were also different between the four groups (*P*<0.01 vs. *P*<0.05, respectively).

**Table 1:** Clinical and metabolic characteristics of study participants according to fasting plasma glucose quartile (n = 697).

FPG	Q1	Q2	Q3	Q4	P value
	(n = 172)	(n = 188)	(n = 199)	(n = 138)	
Mean FPG	77.8 ± 4.2	87.2 ± 2.0	94.7 ± 2.6	107.0 ± 6.2	<0.01
Age (y)	48.9 ± 9.0	50.6 ± 9.1	54.0 ± 8.8	54.2 ± 8.2	<0.01
Men (%)	53.1	60.9	69.1	60.8	<0.01
BMI^1 ^(kg/m^2^)	22.7 ± 2.8	23.2 ± 3.1	23.8 ± 2.7	24.7 ± 2.7	<0.01
SBP^2 ^(mmHg)	117 ± 15	118 ± 14	124 ± 15	127 ± 17	<0.01
DBP^3 ^(mmHg)	73 ± 9	75 ± 9	78 ± 9	80 ± 10	<0.01
Resting heart rate (beats/min)	72 ± 11	73 ± 11	74 ± 11	75 ± 13	<0.05
hs-CRP^4 ^(mg/dL)	0.91 ± 0.87	0.97 ± 1.03	1.07 ± 0.99	1.24 ± 0.98	<0.01
HDL-cholesterol (mg/dL)	54.7 ± 13.3	53.0 ± 12.9	51.0 ± 11.9	49.9 ± 11.6	<0.01
Non-HDL cholesterol (mg/dL)	134.1 ± 34.6	136.7 ± 31.9	144.5 ± 33.0	150.5 ± 38.0	<0.01
Ba-PWV(cm/s)	1303 ± 196	1328 ± 167	1384 ± 216	1469 ± 220	<0.01
Smoker^5 ^(%)	27.9	20.1	23.1	26.1	NS^8^
Alcohol^6 ^(%)	58.1	56.5	62.1	74.0	<0.01
Exercise^7 ^(%)	76.2	71.1	80.0	75.0	NS^8^

Data are shown as the mean ± standard deviation and percentage (%).

*P *values were calculated using the ANOVA and *X*^2^-tests

^1^body mass index; ^2^systolic blood pressure; ^3^diastolic blood pressure; ^4^high sensitivity C-reactive protein; ^5 ^current smokers; ^6^alcohol ingestion≥1 time a week; ^7^regular exercise ≥4 hours a week; ^8^Not significant

**Figure 1:**
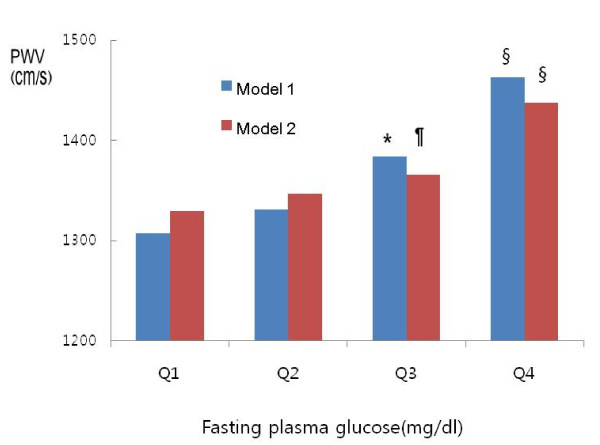
**Mean values of brachial-ankle pulse wave velocity according to fasting plasma glucose quartile in non-diabetic healthy subjects**. *P<0.05 vs. Q1 and Q2, § *P*<0.05 vs. Q1, Q2, and Q3, ¶ *P*<0.05 vs. Q1 Model 1; adjusted for age, sex. Model 2; adjusted for age, sex, systolic blood pressure, diastolic blood pressure, BMI, resting heart rate, Hs-CRP, HDL-cholesterol, and non HDL-cholesterol.

In age-adjusted Pearson's correlations, ba-PWV level was positively correlated with FPG level, systolic blood pressure, diastolic blood pressure, BMI, resting heart rate, hs-CRP, and non-HDL cholesterol, and ba-PWV level was negatively correlated with HDL-cholesterol, as shown in **Table **2. Smoking habits, alcohol ingestion, and exercise were not correlated with ba-PWV. The mean PWV increased according to FPG quartile, as shown in **Table **1. The mean ba-PWV value of the high-normal glucose group (Q3) was higher than those of the low-normal glucose groups (Q1, Q2). Even after adjusting the parameters, the mean ba-PWV in Q3 was higher than that of the low-normal glucose group (Q1). The mean ba-PWV value in the IFG group (Q4) (1438 cm/s) was higher than those of the normal glucose groups (P < 0.05; **Figure **1) after adjustments for age, sex, body mass index, systolic blood pressure, diastolic blood pressure, resting heart rate, HDL-cholesterol, non-HDL cholesterol, and hs-CRP (logarithmically transformed).

**Table 2:** Age-adjusted correlations between brachial-ankle pulse wave velocity and various parameters (n = 697).

	Pulse Wave Velocity	
	
	*R*	*p*-value
FPG^1 ^(mg/dL)	0.278	<0.01
BMI^2 ^(kg/m^2^)	0.089	<0.05
SBP^3 ^(mmHg)	0.502	<0.01
DBP^4 ^(mmHg)	0.472	<0.01
RHR^5^(beats/min)	0.169	<0.01
hs-CRP^6^(mg/dL)	0.167	<0.01
HDL-cholesterol (mg/dL)	-0.146	<0.01
Non-HDL cholesterol (mg/dL)	0.111	<0.01
Current smoking	0.005	NS^7^
Alcohol ingestion	-0.005	NS^7^
Exercise	0.015	NS^7^

Coefficients (*r*) and *p*-values are calculated using the age-adjusted Pearson's correlation model.

^1^fasting plasma glucose; ^2^body mass index; ^3^systolic blood pressure; ^4^diastolic blood pressure; ^5^resting heart rate; ^6 ^high sensitivity C-reactive protein, logarithmically transformed values; ^7^NS, not significant

Through multiple linear regression analysis, ba-PWV was found to be independently and positively associated with FPG, as shown in **Table **3. Ba-PWV was independently and negatively associated with BMI but was not associated with diastolic blood pressure, male: female ratio, HDL-cholesterol, or non-HDL cholesterol.

**Table 3:** Multiple linear regression analyses conducted to assess independent relationships between pulse wave velocity and clinical variables.

Variable	Pulse Wave Velocity	
	
	Regression coefficient (95% CI)	*P *value
FPG	1.40(0.26,2.55)	<0.01
Age	9.60(8.30,10.91)	<0.01
Sex	-21.61(-47.97,4.75)	NS
BMI	-12.34(-17.12,-7.56)	<0.01
SBP	5.24(3.54,6.96)	<0.01
DBP	1.29(-1.56,4.13)	NS
RHR	1.66(0.59,2.72)	<0.01
hs-CRP	15.42(2.01,28.83)	<0.05
HDL-cholesterol	-0.58(-1.59,0.43)	NS
Non-HDL cholesterol	0.23(-0.12,0.57)	NS

*R*^*2*^= 0.48, adjusted *R*^2^= 0.47		

P < 0.001		

FPG, fasting plasma glucose; SBP, systolic blood pressure; DBP, diastolic blood pressure; Sex: male, 1: female, 2; BMI, body mass index; hs-CRP, high-sensitivity C-reactive protein; NS, not significant

## Discussion

In this study, FPG was positively and independently associated with the ba-PWV in non-diabetic healthy adults after correcting for confounding variables. In a recent study, resting heart rate, as an indicator of vascular autonomic balance, was associated with ba-PWV[20]. Therefore, we included resting heart rate as a confounding factor in our analyses. We also found that resting heart rate was associated with the ba-PWV in non-diabetic healthy adults, and FPG was independently associated with ba-PWV after adjusting for resting heart rate and other confounding factors. In a previous study, the ba-PWV level increased from normoglycemic to diabetic subjects. Ohnish *et al*. observed a higher ba-PWV in the IFG than in the normal glucose group, but the authors did not adjust for important confounding factors, such as age, blood pressure, or BMI[13]. Lin Xu *et al*. showed that, when HbA1c was increased, the ba-PWV of normoglycemic subjects, who were grouped according to hemoglobin A1c (HbA1c), increased. However, the authors mentioned that normoglycemic subjects grouped according to FPG did not show the same correlation[21]. Lukich *et al*. found that 284 Caucasian subjects had a positive correlation among FPG, HbA1c, and central artery PWV, and that increased arterial stiffness started at the IFG level according to comparisons of normal glucose, IFG, and diabetes[1].

Based on the difference between the mean ba-PWV values in our study, arterial stiffness may already be occurring in subjects with FPG levels within the normal range[22]. This is the first study to show that patients with high-normal FPG have increased arterial stiffness as measured by ba-PWV. There are some studies that have shown that high normoglycemic status is a risk factor of cardiovascular disease. Twenty-two years of follow-up demonstrated that non-diabetic men with FPG level > 85 mg/dl had a 1.4-fold higher risk of cardiovascular death than did men with lower FPG[23]. In a previous case-controlled study, after excluding patients with diabetes, patients with impaired glucose tolerance and impaired fasting glucose (FPG > 5.2 mmol/l) were found to be 2.7 times (95% CI 1.5- 4.8) more likely to experience myocardial infarction[24]. Our results support the hypothesis that high-normal FPG level is associated with target organ damage and vascular dysfunction and extended the current knowledge by demonstrating a linear, independent relationship between FPG and arterial stiffness in non-diabetic healthy adults.

We excluded six subjects with extremely low FPG level (50-65 mg/dL), even though they had no hypoglycemic symptoms, in order to overcome interpretive difficulty during statistical analysis. These subjects had higher ba-PWV values compared to those of subjects with FPG level between 65-84 mg/dL. We believe that very low FPG does not protect against arterial stiffness, and the relationship between FPG and ba-PWV is a J-shaped curve over the entire range of glucose levels. We suggest that a threshold should be established to avoid arterial stiffness in patients with glucose levels that are within the normal range.

There are some limitations to the generalizability of our results. First, as this study was cross-sectional and was carried out in only one location, it was not possible to establish the exact pathophysiology linking fasting plasma glucose and ba-PWV because we did not consider the characteristics of diverse local environments. Second, up to five percent of subjects with IFG will appear to have diabetes according to 2-hour glucose tolerance testing[25,26]. True-DM and IFG groups were not demarcated in the glucose tolerance test, implying that some people in the IFG group may actually have had DM. Finally, we could not explain the influence of insulin, and further study is needed to adjust for insulin resistance[27,28].

Although this study has some limitations, it is the first attempt to compare arterial stiffness between high-normal FPG (91-100 mg/dL) and low-normal FPG (65-84 mg/dL) subjects as measured by ba-PWV for the purpose of early detection and prevention of arterial stiffness.

## Conclusions

We demonstrated that FPG level, even within the normal range, is associated with aggravation of arterial stiffness in non-diabetic healthy subjects. Further research is needed to determine the optimal glycemic target value for the prevention of arterial stiffness in clinical and public health settings.

## Competing interests

The authors declare that they have no competing interests.

## Authors' contributions

JYS participated in the design of the study, statistical analysis, and preparation of the manuscript. DCL participated in the design of the study and preparation of the manuscript. HRL critically revised the manuscript for important intellectual content, and all authors have given their final approval.
